# Does Number Perception Cause Automatic Shifts of Spatial Attention? A Study of the Att-SNARC Effect in Numbers and Chinese Months

**DOI:** 10.3389/fpsyg.2020.00680

**Published:** 2020-05-12

**Authors:** Dexian He, Xianyou He, Tingting Zhao, Jing Wang, Longzhao Li, Max Louwerse

**Affiliations:** ^1^School of Psychology, South China Normal University, Guangzhou, China; ^2^Center for Studies of Psychological Application, South China Normal University, Guangzhou, China; ^3^Guangdong Key Laboratory of Mental Health and Cognitive Science, South China Normal University, Guangzhou, China; ^4^School of Health Management, Guangzhou Medical University, Guangzhou, China; ^5^Human Resource Department, Guangdong University of Foreign Studies, Guangzhou, China; ^6^Human Resource Department, Architectural Design and Research Institute of Guangdong Province, Guangzhou, China; ^7^Department of Cognitive Science and Artificial Intelligence, Tilburg University, Tilburg, Netherlands

**Keywords:** attention, SNARC effect, mental number line, number processing, ordinal sequences

## Abstract

The Attentional Spatial Numerical Association of Response Codes (Att-SNARC) effect has shown that number perception induces shifts in spatial attention (Fischer et al., 2003; Dodd et al., 2008). However, many replications were attempted and they often failed. In the present study, we investigated whether the Att-SNARC effect can be found for numbers in different notations: months in Arabic form, Simplified Chinese form, Traditional Chinese form (includes numerical ordinal information) and in Chinese non-numerical form (an ordinal sequence). By varying the cognitive task, we also examined whether the effect is a consequence of automatic perceptual processing. In Experiment 1, an Att-SNARC effect was observed for numbers regardless of notation. In Experiment 2 (order-irrelevant task) and Experiment 3 (order-relevant task), the effect was also found consistently for months in Arabic form, Simplified Chinese form, and Traditional Chinese form. This effect was not observed for months in Chinese non-numerical form in Experiment 3. These results show that number and numerical sequence perception automatically causes a spatial shift of attention. Our study provides positive evidence for the Att-SNARC effect and indicates that the effect can generalize to other numerical ordinal sequences that contain numeric information.

## Introduction

There is a strong association between number and space. The spatial-numerical association of response codes (SNARC, Dehaene et al., 1993) effect has been found in a range of studies, showing that when participants make judgments of number magnitude or parity, left-sided response are faster for low-magnitude numbers, whereas right-sided responses are faster for high-magnitude numbers (Fias and Fischer, 2005; Van Dijck et al., 2012). The spatial coding of numbers seems to be automatic (Mapelli et al., 2003; Casarotti et al., 2007). Some researchers believe that these effects can be explained by the putative Mental Number Line (MNL; Restle, 1970; Dehaene et al., 1993; Fischer et al., 2003), a mental representation of number magnitude ordered from left to right in space in which relatively small numbers are associated with left and relatively large numbers with right. According to this view, the effect arises because of the spatial correspondence between the inherent position of the number on the MNL and the position of response keys (Fattorini et al., 2015; Pellegrino et al., 2019). Other researchers claim that reading habits (Dehaene et al., 1993; Shaki et al., 2009; Fischer et al., 2010; Fischer and Brugger, 2011; Göbel et al., 2011) and finger counting (Fischer, 2008; Eerland et al., 2011; Fischer and Brugger, 2011; Lindemann et al., 2011) also contribute to the effect.

Numerous studies regarding space-number associations have been conducted after the classic SNARC effect was found. Fischer et al. (2003) demonstrated that mere observation of numbers causes a shift in covert attention to the left or right side. Perceiving small numbers automatically shifts attention to the left side of space whereas perceiving large numbers automatically shifts attention to the right side of space. This finding was called the Attentional SNARC (Att-SNARC; Fischer et al., 2003; Dodd et al., 2008; Van Dijck et al., 2014) effect. In Table 1, we provide a review of the classic studies on SNARC and Att-SNARC effects.

**TABLE 1:** A review of the classic studies on SNARC and Att-SNARC.

Study	Main research question	Cue type	Tasks and variable delays	Main results
Dehaene et al., 1993	How parity and number magnitude are accessed from Arabic and verbal numerals	Numbers, Letters	Parity judgment task	Large numbers elicited a rightward response and small numbers a leftward response.
Fischer et al., 2003	Whether number perception can induce a shift of attention to the left or right visual field	Numbers	Cue-irrelevant detection task; Variable delay: 50, 100, 200, 300, 400, and 500 ms	Mere observation of numbers automatically activated spatial representations associated with number meaning and caused spatial shifts of attention.
Gevers et al., 2003	Whether non-numerical ordinal sequences are spatially coded	Letters, Months	Order-relevant task; Order-irrelevant task	The automatic SNARC effect was observed for months and letters.
Dodd et al., 2008	Whether numerical and non-numerical ordered sequences share similar mechanisms and would lead to an Att-SNARC effect	Numbers, Letters, Days, Months	Target detection task (order-relevant/order-irrelevant); Variable delay: 250, 500, and 750 ms	The Att-SNARC effect is number-specific and does not automatically generalize to other ordinal sequences.
Zanolie and Pecher, 2014	Whether the Att-SNARC effect is modulated by the relevance of magnitude information	Numbers	Target detection task; Parity judgment task; Magnitude judgment task; Variable delay: 250, 500, 750, and 1,000 ms	The Att-SNARC effect was observed only when participants actively processed number magnitude.
Fattorini et al., 2015	To determine whether the mere perception of numbers causes shifts of spatial attention	Numbers	Target detection task; Parity judgment task; Magnitude judgment tasks; Variable delay: 500, 750 ms	Perceiving numbers does not cause automatic shifts of spatial attention.
Pellegrino et al., 2019	To re-evaluate the consistency and reliability of the Att-SNARC effect	Numbers	Cue-irrelevant detection task; Variable delay: 500, 750 ms	There is no automatic link between the representation of space and the representation of number magnitude.

Many studies have attempted to replicate Fischer et al.’s (2003) finding, but they have had mixed success (Galfano et al., 2006; Ristic et al., 2006; Dodd et al., 2008; Bonato et al., 2009; Van Dijck et al., 2014; Zanolie and Pecher, 2014; Fattorini et al., 2015; Pellegrino et al., 2019). Dodd et al. (2008) extended the Att-SNARC effect and investigated whether it generalizes to other ordinal sequences such as letters, days, and months. They observed an Att-SNARC effect for number stimuli, indicating that numbers can automatically cause spatial shifts of attention. However, the effect was found in ordinal sequences only when participants made an ordinally relevant decision about the stimuli after target detection. Based on these results they concluded that (1) the SNARC effect is sensitive to numerical and non-numeral ordinal stimulus information, whereas the Att-SNARC effect is number-specific; and (2) the SNARC effect reflects response code activation, whereas the Att-SNARC effect reflects changes in visual processing effects due to the allocation of spatial attention.

In a study investigating whether Att-SNARC effects are modulated by the relevance of magnitude information, Zanolie and Pecher (2014) provided mixed results for the idea that perceiving a number induces a shift of visual spatial attention. Spatial representations associated with number meaning were activated and produced a corresponding shift in spatial attention only when participants actively processed number magnitude information. The authors suggested that activation of the MNL is not automatic and might be modulated by the relevance of magnitude information. The type of cognitive processing assigned to numerical cues can influence the presence of the Att-SNARC effect. Indeed, many studies have indicated that SNARC and SNARC-like effects are influenced by the type of cognitive task. Prpic et al. (2016) suggested that information about order and magnitude causing SNARC-like effects may depend on task demands. In their research, participants seemed more likely to process the order of the stimuli in direct tasks and to automatically process the magnitude of the stimuli in indirect tasks. Similarly, Macnamara et al.’s (2018) research showed that the SNARC-like effects were observed when participants performed a direct task, suggesting that the effect is not caused by an automatic process. The SNARC and SNARC-like effects that were observed in direct/relevant tasks were taken as evidence that spatial representations are not automatically activated. Some authors (Galfano et al., 2006; Ristic et al., 2006) have replicated the Att-SNARC effect but have also suggested that it is driven by strategic top-down factors. There is no consensus on whether the Att-SNARC effect can be produced automatically. Moreover, in a recent investigation to reassess the consistency and reliability of the Att-SNARC effect (Fattorini et al., 2015; Pellegrino et al., 2019), results showed no automatic link between the representation of space and the representation of number magnitude.

On the whole, it is unclear from previous studies whether numerical sequences and non-numerical ordinal sequences cause Att-SNARC effects. When effects are produced, is this a consequence of automatic perceptual processes or is this driven by strategic top-down factors?

The SNARC effect is not limited to Arabic numbers, as a similar effect is also elicited by ordinal stimuli such as letters, days, months (Gevers et al., 2003, 2004); non-numerical magnitudes such as the physical size of pictorial surfaces (Prpic et al., 2018); and others such as negative numbers, auditory numbers, or dice patterns (Fischer, 2003; Nuerk et al., 2005). More recently, SNARC and SNARC-like effects have been observed with Chinese characters (Liu et al., 2004, 2011; Hung et al., 2008; Yang et al., 2014; Kopiske et al., 2016; Zhao et al., 2018). To our knowledge, however, there has been only one published report of an Att-SNARC effect for numbers in different notations (Kong et al., 2012), and research has not yet addressed the effect for Chinese months. Therefore, in the current study, we examine the Att-SNARC effect using different notations of numbers and Chinese months as materials.

The present study adopted the attention paradigm used by Dodd et al. (2008) to address two main issues. First, we investigated whether the Att-SNARC effect can be found for numbers, numerical Chinese months, and non-numerical Chinese months regardless of the notation. This should allow us to verify the reliability of the Att-SNARC effect and whether it can generalize to numerical and non-numerical ordinal sequences. Second, by varying the cognitive task, we investigated whether the Att-SNARC effect is a consequence of automatic perceptual processing, or driven by top-down processing. If the Att-SNARC is found in an order-irrelevant task, then it would indicate that the stimuli automatically cause spatial shifts of attention in the visual field. In contrast, if the Att-SNARC effect is observed only when participants are required to make an order-relevant decision, then it would indicate that the effect is influenced by top-down endogenous processes. Addressing these issues should help better understand the cognitive mechanism of the association between number magnitude and special attention.

## Experiment 1

The purpose of Experiment 1 was to investigate whether perceiving numbers in Arabic form, in Simplified Chinese form, and in Traditional Chinese form automatically causes an Att-SNARC effect.

## Methods

### Participants

Thirty undergraduate students were recruited at South China Normal University (22 females, 8 males, mean age = 20.80 ± 2.68 years) and volunteered to participate for an agreed pay of 15 RMB. All students were right-handed Chinese native speakers and naive to the purpose of the experiment. The study was approved by the Institutional Review Board of South China Normal University.

### Materials

The stimulus numbers (one, two, eight, nine) were presented in three forms (see Table 2), including Arabic form [1 (yī), 2 (èr), 8 (bā), 9 (jiǔ)], numbers in Simplified Chinese form [1 (yī), 2 (èr), 8 (bā), 9 (jiǔ)], numbers in Simplified Chinese form [一 (yī), 二 (èr), 八 (bā), 九 (jiǔ)] and numbers in Traditional Chinese form [(壹 (yī), 貳 (èr), 捌 (bā), 玖 (jiǔ)]. Character size was 24 points (Arabic) or 22 points (Chinese). Each numerical value was equally likely to occur in each of the three character types. A 17-inch color 1024 × 768 VGA computer monitor (at 100 Hz) connected to a Pentium IV PC system running E-prime 1.0 was used to present stimuli and record participant responses. A single white text stimulus was presented in the center of the monitor against a black background. The numbers subtended a visual angle of approximately 0.8° in height.

**TABLE 2:** The stimuli used in Experiment 1: numbers in different forms.

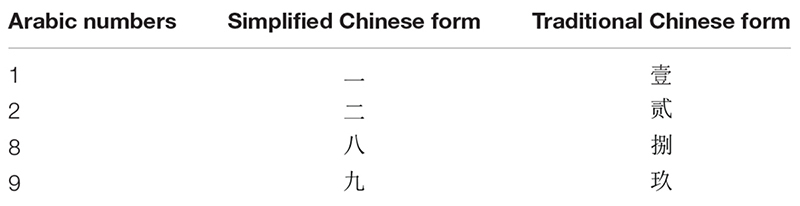

### Procedure

**FIGURE 1:**
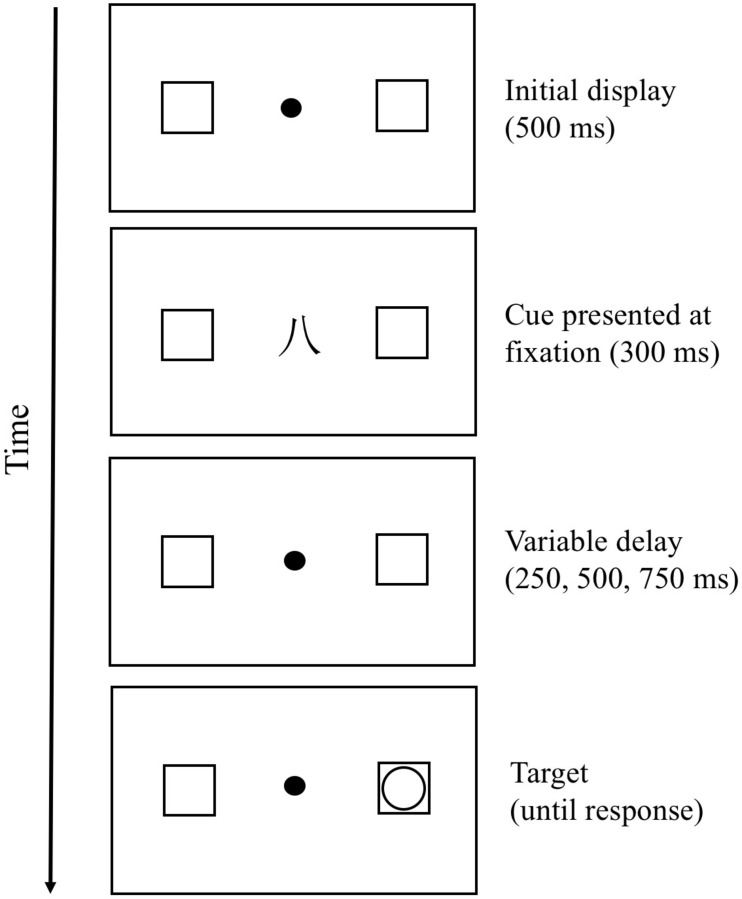
Trial sequence used in Experiment 1.

The present study adopted the same procedure employed by Dodd et al. (2008), except that we used a fixed point “∙” instead of fixation cross “+” to avoid participants regarding the fixation cross “+” as the Simplified Chinese number “十” (which means “ten” in English). The procedure is illustrated in Figure 1. Participants were seated approximately 60 cm from the computer screen. First, a fixation point (white, 0.3° in diameter) was presented for 500 ms, followed by one of three cue types for 300 ms. Before the experiment, participants were told that the cues presented at the fixation were irrelevant and uninformative to target detection. Next, the cue was replaced by a fixation point with a variable stimulus onset asynchrony (SOA) of 250, 500, and 750 ms before target presentation (a white circle subtending 0.8°) so that participants could not anticipate when and where the target would appear. Each type of SOA was equally likely to appear in each trial. Participants were asked to press the space bar as quickly as possible in response to the target appearing in one of two white unfilled squares (each 1° in diameter and 4° to the left and right side of the original fixation point). The probability of target occurrence was equally likely in each square in each trial, and it remained on the screen until the participant responded.

The experiment consisted of three randomized blocks of 288 experimental trials (96 trials in each block). 20 practice trials were administered before three blocks. The only difference between block was the type of cue stimuli presented at the fixation (numbers in Arabic, Simplified Chinese, or Traditional Chinese form). Short breaks were allowed after every 96 trials. The entire task lasted approximately 10–15 minutes.

## Results

**FIGURE 2:**
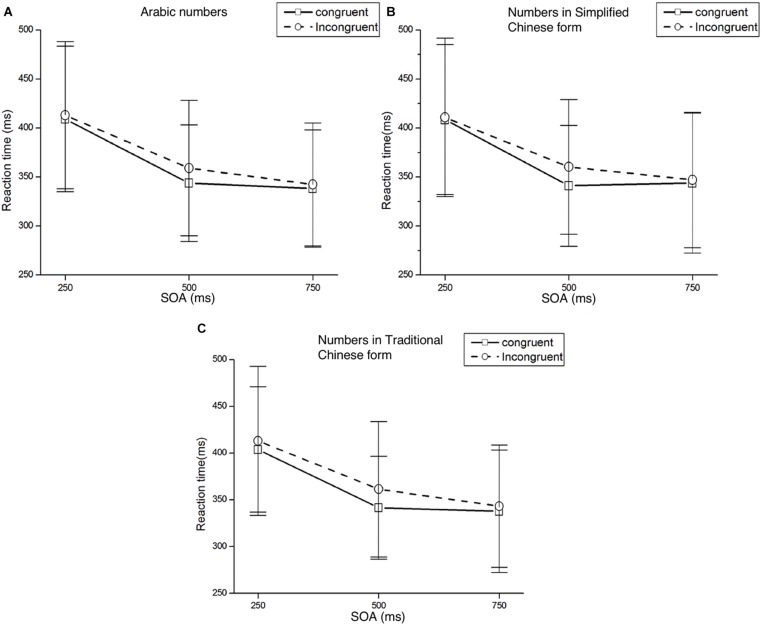
Mean RTs and standard deviations of target detection at each SOA under both congruent and incongruent conditions. Panel **(A)** represents the result of numbers in Arabic form, panel **(B)** represents the result of numbers in Simplified Chinese form, and panel **(C)** represents the result of numbers in Traditional Chinese form.

The analysis was performed according to Dodd et al. (2008). For every participant in each condition, the trials with mean reaction times (RTs) shorter than 100 ms or longer than 1,000 ms were considered errors, accounting for 1.6% of the trials. These data were discarded from subsequent analyses. In Table 3, mean RTs and standard deviations for targets appearing at each target location are presented as a function of cue condition are presented in Table 3. Figure 2 presents the mean RTs and standard deviations of target detection at each SOA under both congruent (i.e., targets appearing on the left when cues were small numbers: 1/一/壹; 2/二/贰) conditions.

**TABLE 3:** Experiment 1-mean RTs (in ms) and standard deviations for targets appearing at each possible location as a function of cue type and SOA.

Cue type		Small numbers (ms)			Large numbers (ms)		
SOA		250	500	750	250	500	750
Numbers in Arabic form	L	410.37 (80.65)	343.88 (59.91)	335.44 (59.22)	406.54 (69.79)	355.14 (69.75)	348.69 (69.26)
	R	419.58 (80.58)	363.13 (69.54)	336.02 (56.03)	408.04 (68.56)	343.60 (60.19)	341.05 (61.30)
Numbers in Simplified Chinese form	L	409.84 (84.30)	336.63 (54.12)	342.62 (80.56)	410.31 (80.83)	362.27 (66.69)	344.64 (71.93)
	R	411.31 (82.15)	358.52 (72.12)	349.37 (67.18)	407.37 (69.23)	345.40 (69.17)	345.13 (62.54)
Numbers in Traditional Chinese form	L	408.05 (69.72)	338.72 (50.03)	335.41 (59.22)	413.01 (81.51)	365.27 (80.72)	348.80 (66.21)
	R	413.14 (79.14)	357.55 (64.38)	337.84 (64.97)	399.66 (65.02)	344.57 (60.68)	340.35 (72.09)

*L represents left target; R represents right target.*

### Numbers in Arabic Form

Mean RTs were analyzed with a 2 (Cue Type: small/large number) × 2 (Target Location: left/right target) × 3 (SOA: 250, 500, 750 ms) ANOVA. There was a significant main effect of SOA, *F*(2,58) = 92.62, *p* < 0.001, η*^2^_*p*_* = 0.762, responses were faster at longer SOAs. There were no other significant main effects of Cue Type or Target Location, *F*(1,29) = 0.08, *p* = 0.786, η*^2^_*p*_* = 0.003 and *F*(1,29) = 0.20, *p* = 0.659, η*^2^_*p*_* = 0.007, respectively. The only other significant effect was the interaction between Cue Type and Target Location, *F*(1,29) = 5.42, *p* = 0.027, η*^2^_*p*_* = 0.158. *Post hoc t*-tests were conducted to determine at which SOAs an effect was present. We found a Att-SNARC effect at the 500 ms SOA for both the left and right target locations: left targets were detected faster than right targets when a small number was presented, *t*(29) = −2.36, *p* < 0.05; right targets were detected faster than left targets when a large number was presented, *t*(29) = 1.75, *p* = 0.09. Results of *post hoc t*-test of Experiment 1 in different SOAs and forms please see Supplementary Table S1.

However, the interaction between Cue Type and SOA was not significant, *F*(2,58) = 1.48, *p* = 0.236, η*^2^_*p*_* = 0.049. The interaction between Target Location and SOA was not significant, *F*(2,58) = 0.73, *p* = 0.485, η*^2^_*p*_* = 0.025. The three-way interaction between Cue Type, Target Location, and SOA was not significant, *F*(2,58) = 1.64, *p* = 0.203, η*^2^_*p*_* = 0.054.

### Numbers in Simplified Chinese Form

Mean RTs were analyzed with a 2 (Cue Type: small/large number) × 2 (Target Location: left/right target) × 3 (SOA: 250, 500, 750 ms) ANOVA. There was a significant main effect of SOA, *F*(2,58) = 81.45, *p* < 0.001, η*^2^_*p*_* = 0.737, with faster responses at longer SOAs. The main effects for Cue Type and Target Location were not significant (*Fs* < 1). As expected, we found a significant interaction effect between Cue Type and Target Location, *F*(1,29) = 4.71, *p* = 0.038, η*^2^_*p*_* = 0.140. *Post hoc t*-tests showed that the Att-SNARC effect was significant at the 500 ms SOA for both the left and right target locations, *t*(29) = -2.09, *p* < 0.05 and *t*(29) = 2.43, *p* < 0.05 respectively.

However, the interaction between Cue Type and SOA was not significant, *F*(2,58) = 0.69, *p* = 0.505, η*^2^_*p*_* = 0. 023. The interaction between Target Location and SOA was not significant, *F*(2,58) = 0.15, *p* = 0.863, η*^2^_*p*_* = 0.005. The three-way interaction between Cue Type, Target Location, and SOA was not significant, *F*(2,58) = 2.52, *p* = 0.089, η*^2^_*p*_* = 0.080.

### Numbers in Traditional Chinese Form

Mean RTs were analyzed with a 2 (Cue Type: small/large number) × 2 (Target Location: left/right target) × 3 (SOA: 250, 500, 750 ms) ANOVA. We found a main effect for SOA, *F*(2,58) = 106.26, *p* < 0.001, η*^2^_*p*_* = 0.786, with faster responses at longer SOAs. The main effects for Cue Type and Target Location were not significant (*Fs* < 1). The only other significant effect was the interaction between Cue Type and Target Location: *F*(1,29) = 6.07, *p* = 0.020, η*^2^_*p*_* = 0.173. *Post hoc t*-test showed a significant Att-SNARC effect at the 500 ms SOA for both the left and right target locations, *t*(29) = -2.10, *p* < 0.05 and *t*(29) = 2.18, *p* < 0.05, respectively.

However, the interaction between Cue Type and SOA was not significant, *F*(2,58) = 1.53, *p* = 0.226, η*^2^_*p*_* = 0.050. The interaction between Target Location and SOA was not significant, *F*(2,58) = 0.08, *p* = 0.924, η*^2^_*p*_* = 0.003. The three-way interaction between Cue Type, Target Location, and SOA was not significant, *F*(2,58) = 2.12, *p* = 0.129, η*^2^_*p*_* = 0.068.

### Interim Discussion

In Experiment 1, an Att-SNARC effect was observed for numbers regardless of the format. Spatial attention was affected by number magnitude. Perceiving small numbers (1/一/壹; 2/二/贰) automatically shifts attention to the left whereas perceiving large numbers (8/八/捌, 9/九/玖) automatically shifts attention to the right. As the effect appeared with all three numerical notations (Arabic, Simplified Chinese, and Traditional Chinese forms), the form of stimulus may not affect the Att-SNARC effect whereas information about magnitude plays an important role in numerical processing during the Att-SNARC effect. The effect is expected to be evoked by the concept instead of the presentation form. In Experiment 2, we further investigated whether the Att-SNARC effect can generalize to other ordinal numerical sequences.

## Experiment 2

The purpose of Experiment 2 was to investigate whether months in different forms can automatically cause an Att-SNARC effect in the same way as numbers.

## Method

### Participants

Thirty undergraduate students were recruited at South China Normal University (20 females, 10 males, mean age = 20.13 ± 2.40 years) and volunteered to participate for an agreed pay of 15 RMB. Participants were different from Experiment 1. All students were right-handed Chinese native speakers and naive to the purpose of the experiment. The study was approved by the Institutional Review Board of South China Normal University.

### Materials

Same as in Dodd et al.’s (2008) research, we chose January, February, August, and September as stimuli (see Table 4). The stimuli were presented in Arabic form [1月(yī yuè), 2月(èr yuè), 8月(bā yuè), 9月(jiǔ yuè)], Simplified Chinese form [一月(yī yuè), 二月(èr yuè), 八月(bā yuè), 九月(jiǔ yuè)], and Traditional Chinese form [壹月(yī yuè), 贰月(èr yuè), 捌月(bā yuè), 玖月(jiǔ yuè)]. To avoid differences in the visual angle of the Arabic months and Chinese numerical months, the Arabic months were presented in Arial font (24 points in size), and the Chinese character month (月) was in boldface (22 points in size). Chinese numerical months were in boldface (22 points in size).

**TABLE 4:** The stimuli used in Experiment 2 and Experiment 3: months in different forms.

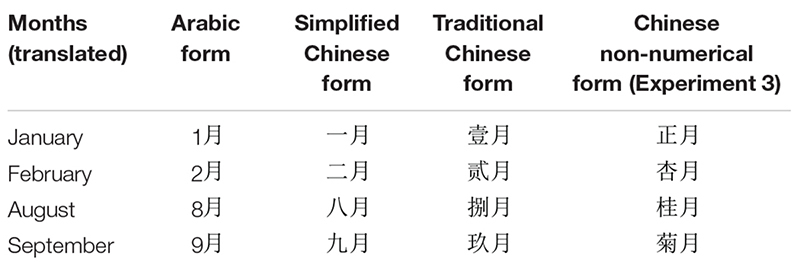

### Procedure

The apparatus and procedure for Experiment 2 were identical to those in Experiment 1. The only difference between these experiments was the type of cue stimuli presented at central fixation.

## Results

**FIGURE 3:**
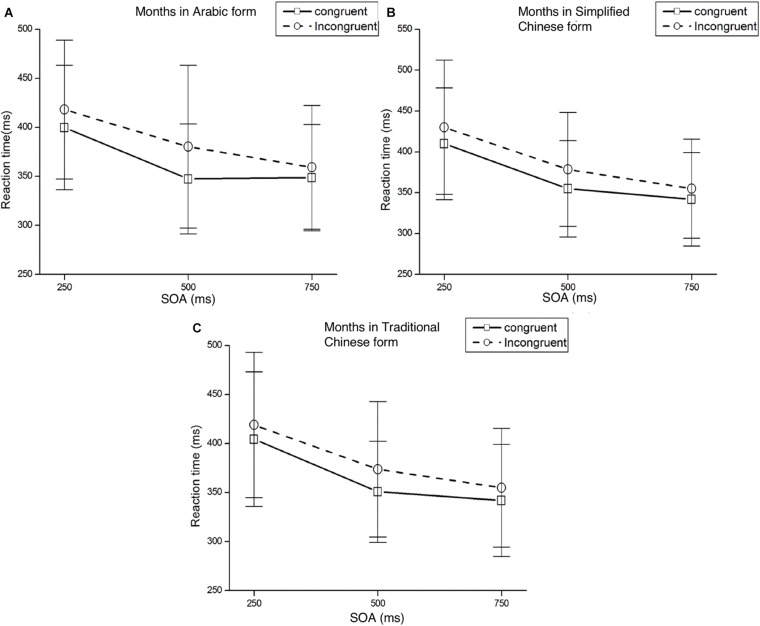
Mean RTs and standard deviations of the target detection at each SOA under both congruent and incongruent conditions. Panel **(A)** represents the result of months in Arabic form, panel **(B)** represents the result of months in Simplified Chinese form, panel **(C)** represents the result of months in Traditional Chinese form.

As in Experiment 1, trials with RTs shorter than 100 ms or longer than 1,000 ms were considered errors, accounting for 2.3% of all trials. These data were discarded from subsequent analyses. In Table 5, mean RTs and standard deviations for targets appearing at each target location are presented as a function of cue condition. Figure 3 presents the mean RTs and standard deviations of target detection at each SOA under both congruent (i.e., targets appearing on the left when cues were months from the beginning of the year: 1月/一月/壹月; 2月/二月/贰月) and incongruent (i.e., targets appearing on the left when cues were months toward the end of the year: 8月/八月/捌月; 9月/九月/玖月) conditions.

**TABLE 5:** Experiment 2–mean RTs (in ms) and standard deviations for targets appearing at each possible location as a function of cue type and SOA.

Cue type		Left months (ms)			Right months (ms)		
SOA		250	500	750	250	500	750
Months in Arabic form	L	396.10 (61.10)	341.66 (53.11)	344.67 (41.55)	424.05 (73.20)	380.27 (87.30)	358.54 (50.25)
	R	412.26 (69.32)	380.35 (80.14)	359.87 (74.96)	403.49 (66.50)	353.18 (59.28)	352.79 (64.98)
Months in Simplified Chinese form	L	406.80 (71.32)	355.72 (51.35)	346.43 (53.81)	435.92 (85.13)	382.00 (73.69)	348.58 (50.72)
	R	424.19 (79.95)	375.09 (67.09)	361.34 (69.37)	413.40 (66.35)	353.96 (66.67)	337.51 (60.88)
Months in Traditional Chinese form	L	408.07 (71.75)	351.39 (52.82)	346.43 (53.81)	423.54 (86.09)	369.33 (66.05)	348.58 (50.72)
	R	414.39 (60.81)	378.37 (72.73)	361.34 (69.37)	400.87 (66.69)	350.45 (51.10)	337.51 (60.88)

### Months in Arabic Form

Mean RTs were analyzed with a 2 (Cue Type: left/right month) × 2 (Target Location: left/right target) × 3 (SOA: 250, 500, 750 ms) ANOVA. There was a significant main effect of SOA, *F*(2,58) = 66.86, *p* < 0.001, η*^2^_*p*_* = 0.797. Participants responded faster in the longer SOA condition. The main effects for Cue Type and Target Location were not significant, *F*(1,29) = 3.44, *p* = 0.074, η*^2^_*p*_* = 0.106 and *F*(1,29) = 0.34, *p* = 0.563, η*^2^_*p*_* = 0.012, respectively. The three-way interaction effect of Cue Type, Target Location, and SOA was significant, *F*(2,58) = 4.27, *p* < 0.05, η*^2^_*p*_* = 0.128. There was a significant interaction effect between Cue Type and Target Location, *F*(1,29) = 5.69, *p* < 0.05, η*^2^_*p*_* = 0.164. *Post hoc t*-tests were conducted to determine at which SOAs the effect was present. A significant Att-SNARC effect was found at the 500 ms SOA for the left and right target location: left targets were detected faster when preceded by months from the beginning of the year, *t*(29) = −2.87, *p* < 0.05; and right targets were detected faster when preceded by months toward the end of the year, *t*(29) = 2.19, *p* < 0.05. Results of *post hoc t*-test of Experiment 2 in different SOAs and forms please see Supplementary Table S2.

However, the interaction between Cue Type and SOA was not significant, *F*(2,58) = 0.44, *p* = 0.643, η*^2^_*p*_* = 0.015. The interaction between Target Location and SOA was not significant, *F*(2,58) = 0.60, *p* = 0.551, η*^2^_*p*_* = 0.020.

### Months in Simplified Chinese Form

Mean RTs were analyzed with a 2 (Cue Type: left/right month) × 2 (Target Location: left/right target) × 3 (SOA: 250, 500, 750 ms) ANOVA. A significant main effect was found for SOA, *F*(2,58) = 64.53, *p* < 0.001, η*^2^_*p*_* = 0.690. The main effects for Cue Type and Target Location were not significant (*Fs* < 1). The only other significant effect was the interaction between Cue Type and Target Location, *F*(1,29) = 6.89, *p* < 0.05, η*^2^_*p*_* = 0.192. *Post hoc t*-tests showed that the Att-SNARC effect was significant at the 500 ms SOA for both the left and right Target Locations, *t*(29) = -2.37, *p* < 0.05 and *t*(29) = 2.35, *p* < 0.05, respectively.

However, the interaction between Cue Type and SOA was not significant, *F*(2,58) = 2.88, *p* = 0.064, η*^2^_*p*_* = 0.090. The interaction between Target Location and SOA was not significant, *F*(2,58) = 0.24, *p* = 0.785, η*^2^_*p*_* = 0.008. The three-way interaction between Cue Type, Target Location, and SOA was not significant, *F*(2,58) = 0.97, *p* = 0.386, η*^2^_*p*_* = 0.032.

### Month in Traditional Chinese Form

Mean RTs were analyzed with a 2 (Cue Type: left/right month) × 2 (Target Location: left/right target) × 3 (SOA: 250, 500, 750 ms) ANOVA. We found a significant main effect of SOA, *F*(2,58) = 65.16, *p* < 0.001, η*^2^_*p*_* = 0.692. The main effects for Cue Type and Target Location were not significant (*Fs* < 1). A significant interaction effect was found between Cue Type and Target Location, *F*(1,29) = 6.12, *p* < 0.05, η*^2^_*p*_* = 0.174. A *post hoc t*-test analysis was conducted, which showed the Att-SNARC effect was significant at the 500 ms SOA for both the left and right target locations, *t*(29) = -2.95, *p* = 0.05 and *t*(29) = 2.29, *p* < 0.05, respectively.

However, the interaction between Cue Type and SOA was not significant, *F*(2,58) = 0.77, *p* = 0.468, η*^2^_*p*_* = 0.026. The interaction between Target Location and SOA was not significant, *F*(2,58) = 1.04, *p* = 0.360, η*^2^_*p*_* = 0.035. The three-way interaction between Cue Type, Target Location, and SOA was not significant, *F*(2,58) = 0.85, *p* = 0.432, η*^2^_*p*_* = 0.028.

In addition, we performed a paired-samples *t*-test comparing Experiment 1 and 2 (see Supplementary Table S3). Mean RTs in Experiment 2 was longer than in Experiment 1 except at the 250 ms SOA for numbers in Arabic form. This is perhaps because numbers convey ordinal information more obviously than months (Dodd et al., 2008). When stimuli convey more salient ordinal information there would be a shorter response time for the activation of a spatial component for the ordinal representation.

## Interim Discussion

In Experiment 2, we found that months in Arabic, Simplified Chinese, and Traditional Chinese forms automatically activate the Att-SNARC effect. Perceiving months from the beginning of the year (1月/一月/壹月; 2月/二月/贰月) shifts attention to the left side of space whereas perceiving months toward the end of the year (8月/八月/捌月, 9月/九月/玖月) shifts attention to the right side of space.

This finding was inconsistent with the results of the study reported by Dodd et al. (2008), in which no Att-SNARC effect was found for ordinal information (months, letters, days) in an order-irrelevant task. This may because English and Chinese month names convey different numerical information. The former is ordinal sequence with no numerical information, while the latter is ordinal numerical sequence for which numerical information still exists. Numbers are frequently used to represent quantity and order in daily life. The spatial representation of numbers is overlearned (Dodd et al., 2008). Therefore, the Att-SNARC effect may have been caused by the additional numerical information presented in Chinese months.

Gevers et al. (2003, 2004) obtained a SNARC effect for ordinal sequences (letters, days, and months) in an order-relevant task, indicating that the mental representation of ordinal sequences is spatially organized. Similarly, Dodd et al. (2008) found that the Att-SNARC effect appeared for ordinal sequences when participants were required to process the cue in an order-relevant fashion. In order to examine whether the Att-SNARC effect generalizes to other ordinal sequences, in Experiment 3 we chose Chinese non-numerical months as stimuli and adopted an order-relevant task.

## Experiment 3

In Experiment 3, we wanted to examine whether an Att-SNARC effect would be observed for numerical and non-numerical ordinal sequence stimuli in an order-relevant task.

## Methods

### Participants

Thirty undergraduate students were recruited at South China Normal University (19 females, 11 males, mean age = 21.31 ± 1.6 years) and volunteered to participate for an agreed pay of 15 RMB. All were right-handed Chinese native speakers and naive to the purpose of the experiment. The study was approved by the Institutional Review Board of South China Normal University.

### Materials

Materials were the same as in Experiment 2, including months in Arabic form [1月(yī yuè), 2月(èr yuè), 8月(bā yuè), 9月(jiǔ yuè)], Simplified Chinese form [一月(yī yuè), 二月(èr yuè), 八月(bā yuè), 九月(jiǔ yuè)], and Traditional Chinese form [壹月(yī yuè), 贰月(èr yuè), 捌月(bā yuè), 玖月(jiǔ yuè)], but also included the ordinal non-numerical form [正月(zhēng yuè), 杏月(xìng yuè), 桂月(guì yuè), 菊月(jú yuè), see Table 4].

### Procedure

The procedure was same as Experiment 2 in Dodd et al.’s (2008), with one exception: when a month appeared at the fixation point, participants were asked whether the month was before or after “May” (i.e., before or after “五月” in the Simplified Chinese form block; before or after “伍月” in the Traditional Chinese form block; and before or after “榴月(liú yuè)” in the non-numerical form block). After a target detection response was made, participants were asked to state aloud whether the cue came before (say “before”) or after (say “after”) May. Each participant completed an experimental session consisting of four randomized blocks of 384 experimental trials. 20 practice trials were administered before four blocks. Short breaks were allowed after every 96 trials. The entire task lasted approximately 15–20 minutes.

## Results

**FIGURE 4:**
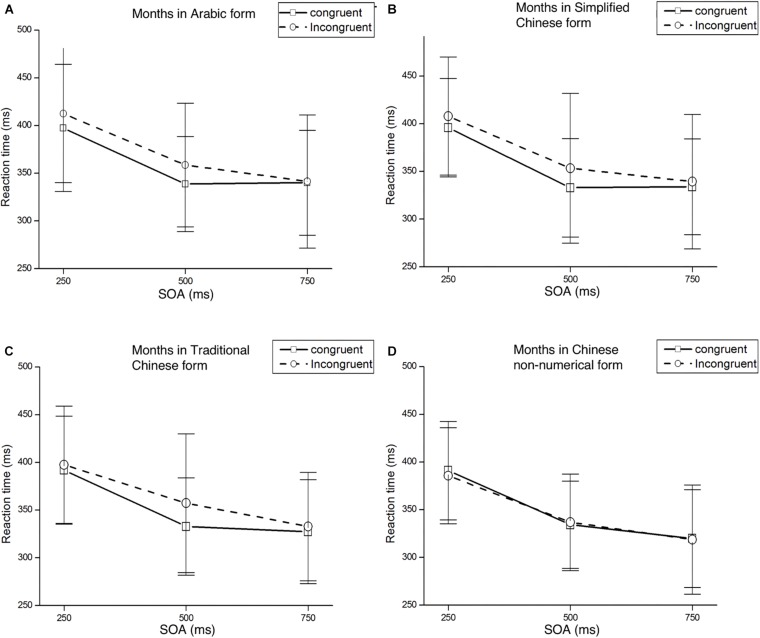
Mean RTs and standard deviations detection at each SOA under both congruent and incongruent conditions. Panel **(A)** represents the result of months in Arabic form, panel **(B)** represents the result of months in Simplified Chinese form, panel **(C)** represents the result of months in Traditional Chinese form, panel **(D)** represents the result of months in Chinese non-numerical form.

Trials with RTs shorter than 100 ms or longer than 1,000 ms were again considered errors, accounting for 3.7% of all trials. These data were discarded from subsequent analyses. In Table 6, mean RTs and standard deviations for targets appearing at each target location are presented as a function of cue condition. Figure 4 presents mean RTs and standard deviations of target detection at each SOA under both congruent and incongruent conditions.

**TABLE 6:** Experiment 3-mean RTs (in ms) and standard deviations for targets appearing at each possible location as a function of cue type and SOA.

Cue type		Left months (ms)			Right months (ms)		
SOA		250	500	750	250	500	750
Months in Arabic form	L	398.38 (67.21)	337.06 (45.08)	344.50 (58.84)	414.58 (76.31)	356.72 (60.66)	336.80 (64.00)
	R	410.48 (70.87)	360.77 (70.84)	346.18 (76.82)	396.43 (68.30)	340.41 (55.80)	335.78 (52.60)
Months in Simplified Chinese form	L	396.59 (53.34)	334.46 (51.95)	323.92 (50.49)	409.34 (68.45)	349.43 (80.49)	347.67 (82.79)
	R	406.70 (55.78)	357.40 (77.65)	331.00 (55.62)	395.33 (50.74)	331.45 (52.06)	343.90 (48.82)
Months in Traditional Chinese form	L	385.46 (57.59)	333.73 (49.48)	335.31 (64.56)	401.85 (76.01)	353.85 (75.54)	325.53 (45.00)
	R	393.27 (43.13)	360.77 (70.84)	340.17 (66.60)	398.49 (55.75)	331.93 (53.44)	319.62 (41.60)
Months in Chinese non-numerical form	L	393.03 (53.80)	342.08 (48.07)	320.04 (59.61)	386.22 (51.26)	331.89 (44.33)	325.19 (54.39)
	R	385.09 (50.23)	341.89 (56.63)	312.34 (60.22)	389.15 (50.08)	326.59 (42.62)	319.72 (42.35)

### Months in Arabic Form

Mean RTs were analyzed with a 2 (Cue Type: left/right month) × 2 (Target Location: left/right target) × 3 (SOA: 250, 500, 750 ms) ANOVA. There was a significant main effect of SOA, *F*(2,58) = 50.04, *p* < 0.001, η*^2^_*p*_* = 0.633, with faster responses in the longer SOA condition. There were no other significant main effects of Cue Type or Target Location, *F*(1,29) = 0.78, *p* = 0.385, η*^2^_*p*_* = 0.026 and *F*(1,29) = 0.06, *p* = 0.938, η*^2^_*p*_* = 0.00, respectively. The three-way interaction between Cue Type, Target Location, and SOA was significant, *F*(2,58) = 3.43, *p* < 0.05, η*^2^_*p*_* = 0.106. The interaction between the Cue Type and Target Location was significant, *F*(1,29) = 4.98, *p* < 0.05, η*^2^_*p*_* = 0.15. *Post hoc t*-tests showed that the Att-SNARC effect was significant at the 500 ms SOA for both the left and right target locations: left targets were detected faster when preceded by months from the beginning of the year, *t*(29) = −1.95, *p* = 0.06; right targets were detected faster when preceded by months toward the end of the year, *t*(29) = 2.57, *p* < 0.05. Results of *post hoc t*-test of Experiment 3 in different SOAs and forms please see Supplementary Table S4.

However, the interaction between Cue Type and SOA was not significant, *F*(2,58) = 0.53, *p* = 0.594, η*^2^_*p*_* = 0.018. The interaction between Target Location and SOA was not significant, *F*(2,58) = 0.40, *p* = 0.675, η*^2^_*p*_* = 0.013.

### Months in Simplified Chinese Form

Mean RTs were analyzed with a 2 (Cue Type: left/right month) × 2 (Target Location: left/right target) × 3 (SOA: 250, 500, 750 ms) ANOVA. There was a significant main effect of SOA, *F*(2,58) = 54.68, *p* < 0.001, η*^2^_*p*_* = 0.65. There were no other significant main effects of the Cue Type [*F*(1,29) = 1.06, *p* = 0.312, η*^2^_*p*_* = 0.035] or Target Location [*F*(1,29) = 0.03, *p* = 0.865, η*^2^_*p*_* = 0.001]. A significant interaction effect was found between the Cue Type and Target Location, *F*(1,29) = 5.45, *p* < 0.05, η*^2^_*p*_* = 0.158. *Post hoc t*-test showed a Att-SNARC effect at the 500 ms SOA for both the left and right target locations, *t*(29) = -2.09, *p* < 0.05 and *t*(29) = 1.78, *p* = 0.085, respectively. In addition, a significant interaction effect was found between the Cue Type and SOA, *F*(2,58) = 4.48, *p* < 0.05, η*^2^_*p*_* = 0.134.

However, the interaction between Target Location and SOA was not significant, *F*(2,58) = 0.17, *p* = 0.841, η*^2^_*p*_* = 0.006. The three-way interaction between Cue Type, Target Location, and SOA was also not significant, *F*(2,58) = 1.85, *p* = 0.167, η*^2^_*p*_* = 0.060.

### Months in Traditional Chinese Form

Mean RTs were analyzed with a 2 (Cue Type: left/right month) × 2 (Target Location: left/right target) × 3 (SOA: 250, 500, 750 ms) ANOVA. A significant main effect for SOA was found, *F*(2,58) = 67.61, *p* < 0.001, η*^2^_*p*_* = 0.700. There were no other significant main effects of Cue Type [*F*(1,29) = 0.57, *p* = 0.457, η*^2^_*p*_* = 0.019] or Target Location [*F*(1,29) = 0.13, *p* = 0.722, η*^2^_*p*_* = 0.004]. The only other significant effect was the interaction between the Cue Type and Target Location, *F*(1,29) = 4.93, *p* < 0.05, η*^2^_*p*_* = 0.145. We found a significant Att-SNARC effect at the 500 ms SOA for both the left and right target locations using a *post hoc t*-test analysis, *t*(29) = -2.19, *p* < 0.05 and *t*(29) = 2.30, *p* < 0.05, respectively.

However, the interaction between Target Location and SOA was not significant, *F*(2,58) = 0.05, *p* = 0.954, η*^2^_*p*_* = 0.002. The interaction between Cue Type and SOA was not significant, *F*(2,58) = 2.46, *p* = 0.095, η*^2^_*p*_* = 0.078. The three-way interaction between the Cue Type, Target Location, and SOA was also not significant, *F*(2,58) = 2.86, *p* = 0.065, η*^2^_*p*_* = 0.090.

### Months in Chinese Non-numerical Ordinal Form

Mean RTs were analyzed with a 2 (Cue Type: left/right month) × 2 (Target Location: left/right target) × 3 (SOA: 250, 500, 750 ms) ANOVA. A main effect of SOA again appeared in this analysis, *F*(2,58) = 85.14, *p* < 0.001, η*^2^_*p*_* = 0.746, with faster responses in the longer SOA condition. However, there were no significant main effects of the Cue Type or Target Location, *F*(1,29) = 0.22, *p* = 0.643, η*^2^_*p*_* = 0.008 and *F*(1,29) = 1.43, *p* = 0.241, η*^2^_*p*_* = 0.047, respectively. The interaction between Cue Type and Target Location was not significant, *F*(1,29) = 2.33, *p* = 0.633, η*^2^_*p*_* = 0.008. The interaction between the Cue Type and SOA was not significant, *F*(2,58) = 2.71, *p* = 0.075, η*^2^_*p*_* = 0.085. The interaction between the Target Location and SOA was not significant, *F*(2,58) = 0.18, *p* = 0.837, η*^2^_*p*_* = 0.006. The three-way interaction between the Cue Type, Target Location, and SOA was also not significant, *F*(2,58) = 0.43, *p* = 0.671, η*^2^_*p*_* = 0.015.

## Interim Discussion

In Experiment 3, an Att-SNARC effect was observed for months in Arabic, Simplified Chinese, and Traditional Chinese forms; however, the effect was not observed for months in Chinese non-numerical form. A possible explanation for this result is that months in Chinese non-numerical form are an ordinal non-numerical sequence without numerical properties. Besides, it is worth noting that the three-way interaction was significant only for months in Arabic form in Experiment 2 and Experiment 3. Three-way interaction between Cue Type, Target Location, and SOA indicates Att-SNARC modulated by SOA, whereas two-way interaction between Cue Type, Target Location indicates a general Att-SNARC.

In addition, we compared mean RTs in Experiment 2 and Experiment 3 (see Supplementary Table S5). The results show that RTs are faster in the order-relevant task than in the order-irrelevant task. Specifically, for the months in Arabic form and Traditional Chinese form, mean RTs in Experiment 3 were significantly shorter than in Experiment 2 at the 500 ms SOA, *t*(119) = −2.076, *p* = 0.040 and *t*(119) = -3.147, *p* = 0.002, respectively. This response difference could be explained by greater attention to the cue’s magnitude causing faster target detection. Spotlight (Posner et al., 1980) and zoom lens (Eriksen and St. James, 1986) models of spatial attention suggest that attention influences the speed of processing in the visual system (Casarotti et al., 2007). Participants use more attentional resources for deeper processing tasks than shallow processing tasks. The explicit processing of magnitude causes more attention to magnitude information of the cue and the activation of spatial representations associated with ordinal meaning, thereby increasing processing efficiency of target detection.

## General Discussion

In this study, we investigated whether the Att-SNARC effect can be found in numbers and other numerical and non-numerical ordinal sequences (Chinese months). Some authors claim that number perception induces a spatial shift of attention (Fischer et al., 2003; Galfano et al., 2006; Ristic et al., 2006; Casarotti et al., 2007; Dodd et al., 2008). Dodd et al. (2008) suggest that the effect can generalize to non-numerical ordinal sequences when participants are required to process magnitude information in an order-relevant fashion. Some authors have failed to replicate the Att-SNARC, or observed the effect for numbers only when participants actively processed number magnitude (Van Dijck et al., 2014; Zanolie and Pecher, 2014; Fattorini et al., 2015; Pellegrino et al., 2019). In light of the mixed results reported in previous studies, we ran three experiments using Arabic numbers (Experiment 1), the months in Arabic, Simplified Chinese, and Traditional Chinese forms (Experiment 2), and the months in Chinese non-numerical form (Experiment 3, order-relevant task). The main results show that perception of numbers and other numerical ordinal sequence (months in Arabic, Simplified Chinese, and Traditional Chinese forms) presented at a central fixation produce automatic magnitude-related shifts of spatial attention. However, the Att-SNARC effect is specific to numerical sequence processing and does not generalize to non-numerical ordinal sequences.

In Experiment 1, we aimed to determine whether the mere perception of numbers at a central fixation causes automatic shifts of spatial attention by using numbers in Arabic, Simplified Chinese, and Traditional Chinese forms. The Att-SNARC effect was found for all number formats at the 500 ms SOA: targets were detected faster in the left side of space than in the right when a small number (e.g., 1/一/壹, 2/二/贰) was presented; targets were detected faster in the right side of space than in the left when a large number (e.g., 8/八/捌, 9/九/玖) was presented. The associations between numbers and spatial representations are modality-independent. Regardless of format, number perception automatically activates a spatial representation associated with magnitude and causes a shift of spatial attention.

In Experiment 2, our goal was to further investigate whether the Att-SNARC effect can be observed in other numerical ordinal sequences. The results show that a significant Att-SNARC effect was found at the 500 ms SOA: targets in the left side of space were detected faster when preceded by months from the beginning of the year (e.g., 1月/一月/壹月, 2月/二月/贰月); targets in the right side of space were detected faster when preceded by months toward the end of the year (e.g., 8月/八月/捌月, 9月/九月/玖月). The results are partially inconsistent with the Dodd et al.’s (2008) findings that an Att-SNARC effect was observed for months only when the participants were required to process the cue in an order-relevant fashion. This is perhaps because months in Arabic, Simplified Chinese, and Traditional Chinese forms all contain numerical information, unlike the months used in Dodd et al.’s (2008) study. Left-to-right representations of number magnitude can be elicited when processing numerical month stimuli. Thus, these materials produce similar effects as numbers.

Converging evidence from Experiment 1 and 2 suggests that the Att-SNARC effect can be elicited by numbers and other numerical ordinal sequences. The association between shifts of spatial attention and number magnitude is automatic, not driven by strategic top-down processing. These results suggest that a similar processing mechanism might exist for numbers and numerical Chinese months (months in Arabic, Simplified Chinese, and Traditional Chinese forms).

In Experiment 3 (order-relevant task), we tested the same stimuli used in Experiment 2 and added non-numerical ordinal stimuli (e.g., 正月, 杏月, 桂月, 菊月). An Att-SNARC effect was again observed in numerical ordinal sequences (months in Arabic, Simplified Chinese, and Traditional Chinese forms). It is possible that the Att-SNARC effect observed in our experiments is mainly influenced by the numerical prefix. However, we did not find the Att-SNARC effect in Chinese non-numerical months, indicating that the effect does not generalize to ordinal sequences, even in an order-relevant task.

One possible explanation for these results is that there is a tight link between space and numbers due to the influences of culture and experience. Reading habits and finger counting habits provide importance contributions to the left-to-right organization of the MNL and the occurrence of SNARC effects (Dehaene et al., 1993; Fischer, 2008; Shaki et al., 2009; Fischer et al., 2010; Eerland et al., 2011; Fischer and Brugger, 2011; Göbel et al., 2011; Lindemann et al., 2011). People frequently use numbers in real life situations to represent quantity and order. Consequently, a tight association between space and numbers is established in which left/right spatial codes are linked to small/large number magnitudes. These culturally acquired and spatially meaningful stimuli automatically produce magnitude-related shifts of spatial attention in target detection tasks. However, Chinese non-numerical ordinal months (e.g., 正月, 杏月, 桂月, 菊月) mainly appear in poetry and are less frequently used in contemporary China. Due to the unfamiliarity of Chinese non-numerical ordinal months, the association between the magnitude of these stimuli and space is too weak to evoke the Att-SNARC effect.

Another possibility is that Chinese numerical and non-numerical months have different properties. The former are numerical ordinal stimuli that contain numeral information (e.g., 1月, which means January), whereas the latter is a non-numerical ordered sequence (e.g., 正月, which means January) similar to days, letters, and English months. Numbers convey ordinal information in a more salient manner than an ordinal sequence (Dodd et al., 2008). Participants mainly encode numerical information when processing numbers and numerical Chinese months. Therefore, the left-to-right spatial coding of number and/or ordinal magnitudes is activated. However, due to the properties of a non-numerical ordinal sequence, the strength and reliability of a spatial association might not be as strong as with numbers. Participants might process ordinal information when perceiving non-numerical month stimuli. For instance, “菊月(September)” is after “榴月(May)” and “杏月(February)” is the second month of the year. They might also associate non-numerical months with other semantic information irrelevant to space. For instance, “杏月(February)” might evoke “春天 (spring)” or “杏 (apricot).” This irrelevant information might interfere with activation of a spatial component of the ordinal representation. Therefore, the Att-SNARC effect would only be observed in numbers and numerical ordinal months.

The results of our study also showed that the cognitive mechanisms of the Att-SNARC effect are different from the SNARC effect. The SNARC effect was found in different notations, including letters, days, months, auditory number word, visual Arabic numeral, visual number word, and visual dice pattern. This indicates that the SNARC effect is modality-independent (Gevers et al., 2003, 2004; Nuerk et al., 2005). Motor responses in left-to-right space might play an important role in accessing spatial codes of magnitude related information, thus causing a consistent SNARC effect in different notation conditions. Nonetheless, our study suggested that the Att-SNARC effect is only sensitive to numbers and ordinal sequence that contain numeric information.

In summary, the findings from our study provide evidence that perceiving numbers causes an automatic shift of spatial attention. We replicated and extended partial results from previous research. The Att-SNARC effect is not just number-specific. It can also be observed in some numerical ordinal sequences, e.g., months in Arabic, Simplified Chinese, and Traditional Chinese forms. To our knowledge, the present study is the first to examine the Att-SNARC effect in different forms of Chinese months. However, there are noteworthy limitations in the current research. First, we did not examine the Att-SNARC effect in non-numerical Chinese months in Experiment 2. Therefore, we were unable to directly compare the performance difference between order-irrelevant and order-relevant tasks. Second, the present experiments did not include catch trials (false alarms). We believe that future studies would benefit from the addition of catch trials, which may help to estimate the level at which a participant is guessing when no target is present. In addition, future studies are needed to systematically determine the cognitive mechanisms underlying the perception of numbers and numerical Chinese months.

## Data Availability Statement

The datasets generated for this study are available on request to the corresponding author.

## Ethics Statement

The studies involving human participants were reviewed and approved by Human Research Ethics Committee for Non-Clinical Faculties, School of Psychology, South China Normal University. The participants provided their written informed consent to participate in this study.

## Author Contributions

DH analyzed the data and wrote and revised the manuscript. XH and ML contributed to the conception of the study and manuscript revisions. TZ analyzed the data and wrote the manuscript. JW and LL designed the study and collected the data. All authors approved the final version of the manuscript.

## Conflict of Interest

The authors declare that the research was conducted in the absence of any commercial or financial relationships that could be construed as a potential conflict of interest.
