# Supplementary material for: Does Number Perception Cause Automatic Shifts of Spatial Attention? A Study of the Att-SNARC Effect in Numbers and Chinese Months
Source: Front Psychol. 2020 May 12;11:680. doi: 10.3389/fpsyg.2020.00680 (PMC7235174; doi:10.3389/fpsyg.2020.00680)
Supplement: Supplementary file 1 [file Data_Sheet_1.docx]

**Supplementary Materials**

**Table S1** Results of *post hoc* *t*-test of Experiment 1

| **SOA** | **Cue Type** | **Arabic form** | | **Simplified Chinese form** | | **Traditional Chinese form** | |
| --- | --- | --- | --- | --- | --- | --- | --- |
|  |  | *t*(29) | Sig. | *t*(29) | Sig. | *t*(29) | Sig. |
| 250 ms | S | -0.952 | 0.349 | -0.163 | 0.872 | -0.607 | 0.549 |
|  | L | -0.185 | 0.854 | 0.373 | 0.712 | 1.390 | 0.175 |
| 500 ms | S | -2.359* | 0.025 | -2.086 | 0.046 | -2.095* | 0.045 |
|  | L | 1.753 | 0.090 | 2.431 | 0.021 | 2.182* | 0.037 |
| 750 ms | S | -0.073 | 0.942 | -0.683 | 0.500 | -0.310 | 0.759 |
|  | L | 0.892 | 0380 | -0.077 | 0.939 | 1.218 | 0.233 |

*Note. Dependent variable is mean response time; **p<0.01, *p<0.05; S represents small number; L represents large number.*

**Table S2** Results of *post hoc t*-test of Experiment 2

| **SOA** | **Cue Type** | **Arabic form** | | **Simplified Chinese form** | | **Traditional Chinese form** | |
| --- | --- | --- | --- | --- | --- | --- | --- |
|  |  | *t*(29) | Sig. | *t*(29) | Sig. | *t*(29) | Sig. |
| 250 ms | L | -1.441 | 0.160 | -1.398 | 0.173 | -0.575 | 0.570 |
|  | R | 1.845 | 0.75 | 1.966 | 0.059 | 1.962 | 0.059 |
| 500 ms | L | -2.872** | 0.008 | -2.366 | 0.025 | -2.945** | 0.006 |
|  | R | 2.190* | 0.037 | 2.346 | 0.026 | 2.290* | 0.029 |
| 750 ms | L | -1.246 | 0.223 | -1.092 | 0.284 | -1.092 | 0.284 |
|  | R | 0.594 | 0.557 | 1.490 | 0.147 | 1.490 | 0.147 |

*Note. Dependent variable is mean response time; **p<0.01, *p<0.05; L represents left month (months from the beginning of the year); R represents right month (months towards the end of the year).*

**Table S3** Results of paired-samples *t*-test of Experiment 2 and Experiment 1 in different SOAs and forms

| **SOA** | **Arabic form** | | **Simplified Chinese**  **form** | | **Traditional Chinese**  **form** | |
| --- | --- | --- | --- | --- | --- | --- |
|  | *t*(119) | Sig. | *t*(119) | Sig. | *t*(119) | Sig. |
| 250 ms | -0.264 | 0.792 | 0.411 | 0.682 | 1.185 | 0.238 |
| 500 ms | 1.556 | 0.122 | 1.440 | 0.153 | 2.197* | 0.030 |
| 750 ms | 1.922 | 0.057 | 1.093 | 0.276 | 0.392 | 0.696 |

*Note. Dependent variable is mean response time; **p<0.01, *p<0.05*

**Table S4** Results of *post hoc* *t*-test of Experiment 3

| **SOA** | **Target Type** | **Arabic form** | | **Simplified Chinese form** | | **Traditional Chinese form** | | **Non-numerical form** | | |
| --- | --- | --- | --- | --- | --- | --- | --- | --- | --- | --- |
|  |  | *t*(29) | Sig. | *t*(29) | Sig. | *t*(29) | Sig. | *t*(29) | Sig. |  |
| 250 ms | L | -1.522 | 0.139 | -1.212 | 0.235 | -0.856 | 0.399 | 1.000 | 0.325 |  |
|  | R | 1.914 | 0.066 | 1.537 | 0.135 | 0.272 | 0.787 | -0.296 | 0.769 |  |
| 500 ms | L | -1.952 | 0.061 | -2.088* | 0.046 | -2.192* | 0.037 | 0.025 | 0.980 |  |
|  | R | 2.571* | 0.016 | 1.781 | 0.085 | 2.301* | 0.029 | 0.728 | 0.472 |  |
| 750 ms | L | -0.142 | 0.888 | -1.328 | 0.195 | -0.422 | 0.676 | 1.152 | 0.259 |  |
|  | R | 0.176 | 0.862 | 0.329 | 0.744 | 0.748 | 0.461 | 0.724 | 0.475 |  |

*Note. Dependent variable is mean response time; **p<0.01, *p<0.05; L represents left month (months from the beginning of the year); R represents right month (months towards the end of the year).*

**Table S5** Results of paired-samples *t*-test of Experiment 3 and Experiment 2 in different SOAs and forms

| **SOA** | **Arabic form** | | **Simplified Chinese**  **form** | | **Traditional Chinese**  **form** | |
| --- | --- | --- | --- | --- | --- | --- |
|  | *t*(119) | Sig. | *t*(119) | Sig. | *t*(119) | Sig. |
| 250 ms | -0.497 | 0.620 | -2.223* | 0.028 | -2.630** | 0.010 |
| 500 ms | -2.076* | 0.040 | -2.547 | 0.012 | -3.147** | 0.002 |
| 750 ms | -1.911 | 0.058 | -2.676** | 0.008 | -1.662 | 0.099 |

*Note. Dependent variable is mean response time; **p<0.01, *p<0.05*
